# Maxizyme-mediated suppression of chikungunya virus replication and transmission in transgenic *Aedes aegypti* mosquitoes

**DOI:** 10.3389/fmicb.2023.1286519

**Published:** 2023-12-22

**Authors:** Priya Mishra, Velmurugan Balaraman, Malcolm J. Fraser

**Affiliations:** Department of Biological Sciences, Eck Institute for Global Health, University of Notre Dame, Notre Dame, IN, United States

**Keywords:** maxizyme (Mz), hammerhead ribozyme (hRz), chikungunya (CHIKV), connected maxizyme (cMz), mosquito transmission

## Abstract

Chikungunya virus (CHIKV) is an emerging mosquito-borne pathogen of significant public health importance. There are currently no prophylactic vaccines or therapeutics available to control CHIKV. One approach to arbovirus control that has been proposed is the replacement of transmission-competent mosquitoes with those that are refractory to virus infection. Several transgene effectors are being examined as potentially useful for this population replacement approach. We previously demonstrated the successful use of hammerhead ribozymes (hRzs) as an antiviral effector transgene to control CHIKV infection of, and transmission by, Aedes mosquitoes. In this report we examine a maxizyme approach to enhance the catalytic activity and prevent virus mutants from escaping these ribozymes. We designed a maxizyme containing minimized (monomer) versions of two hRzs we previously demonstrated to be the most effective in CHIKV suppression. Three versions of CHIKV maxizyme were designed: Active (Mz), inactive (ΔMz), and a connected CHIKV maxizyme (cMz). The maxizymes with their expression units (Ae-tRNA ^val^ promoter and its termination signal) were incorporated into lentivirus vectors with selection and visualization markers. Following transformation, selection, and single-cell sorting of Vero cells, clonal cell populations were infected with CHIKV at 0.05 and 0.5 MOI, and virus suppression was assessed using TCID_50_-IFA, RT-qPCR, and caspase-3 assays. Five transgenic mosquito lines expressing cMz were generated and transgene insertion sites were confirmed by splinkerette PCR. Our results demonstrate that Vero cell clones expressing Mz exhibited complete inhibition of CHIKV replication compared to their respective inactive control version or the two parent hRzs. Upon oral challenge of transgenic mosquitoes with CHIKV, three out of the five lines were completely refractory to CHIKV infection, and all five lines tested negative for salivary transmission. Altogether, this study demonstrates that maxizymes can provide a higher catalytic activity and viral suppression than hRzs.

## Introduction

Chikungunya virus (CHIKV) is an enveloped, single-stranded, positive-sense RNA virus that belongs to the genus *Alphavirus* and family *Togaviridae*. CHIKV is transmitted to humans by *Aedes aegypti* and *Ae. albopictus* mosquitoes (Weaver and Lecuit, 2015; Higgs and Vanlandingham, 2018), causing chikungunya fever (CF), which is characterized by symptoms such as fever, myalgia, and debilitating joint pain that may last for months (Pialoux et al., 2007; Weaver and Lecuit, 2015; Halstead, 2018), and in some cases, can result in fatality (Cardona-Ospina et al., 2015). The disease can impact the economy in several spheres, significantly affecting the health system and national economies (Costa et al., 2023). Currently there are no effective prophylactic or therapeutic measures to control CF, although a putative vaccine is making its way through clinical trials (Schmidt and Schnierle, 2022; Schneider et al., 2023).

Since arboviruses such as CHIKV require mosquitoes to complete their transmission cycle (Franz et al., 2006), alternative approaches aimed at reducing or replacing naturally competent mosquitoes with virus refractory mosquitoes expressing various transgene effectors have been pursued to control arboviruses (Gantz et al., 2015; Aliota et al., 2016; Williams et al., 2020; Reid et al., 2021).

In our lab we have been exploring the potential of several types of antiviral ribozymes including hammerhead ribozyme (hRz) and maxizyme (Mz), among others (Nawtaisong et al., 2009; Carter et al., 2015; Mishra et al., 2016). Hammerhead ribozymes are small catalytic RNA molecules that can cleave target RNA in a sequence-specific manner. In contrast, maxizymes (Mz) are dual-catalytic RNA molecules capable of simultaneously cleaving multiple target sequences in an RNA molecule.

We previously identified two hRzs, #9 and #14, targeting the sub-genomic region of CHIKV that were effective at inhibiting CHIKV 181/25 replication both *in vitro* and *in vivo* (Mishra et al., 2016). However, these single hRzs target regions of 19 nt in size, making them potentially susceptible to escape variants.

**Figure 1:**
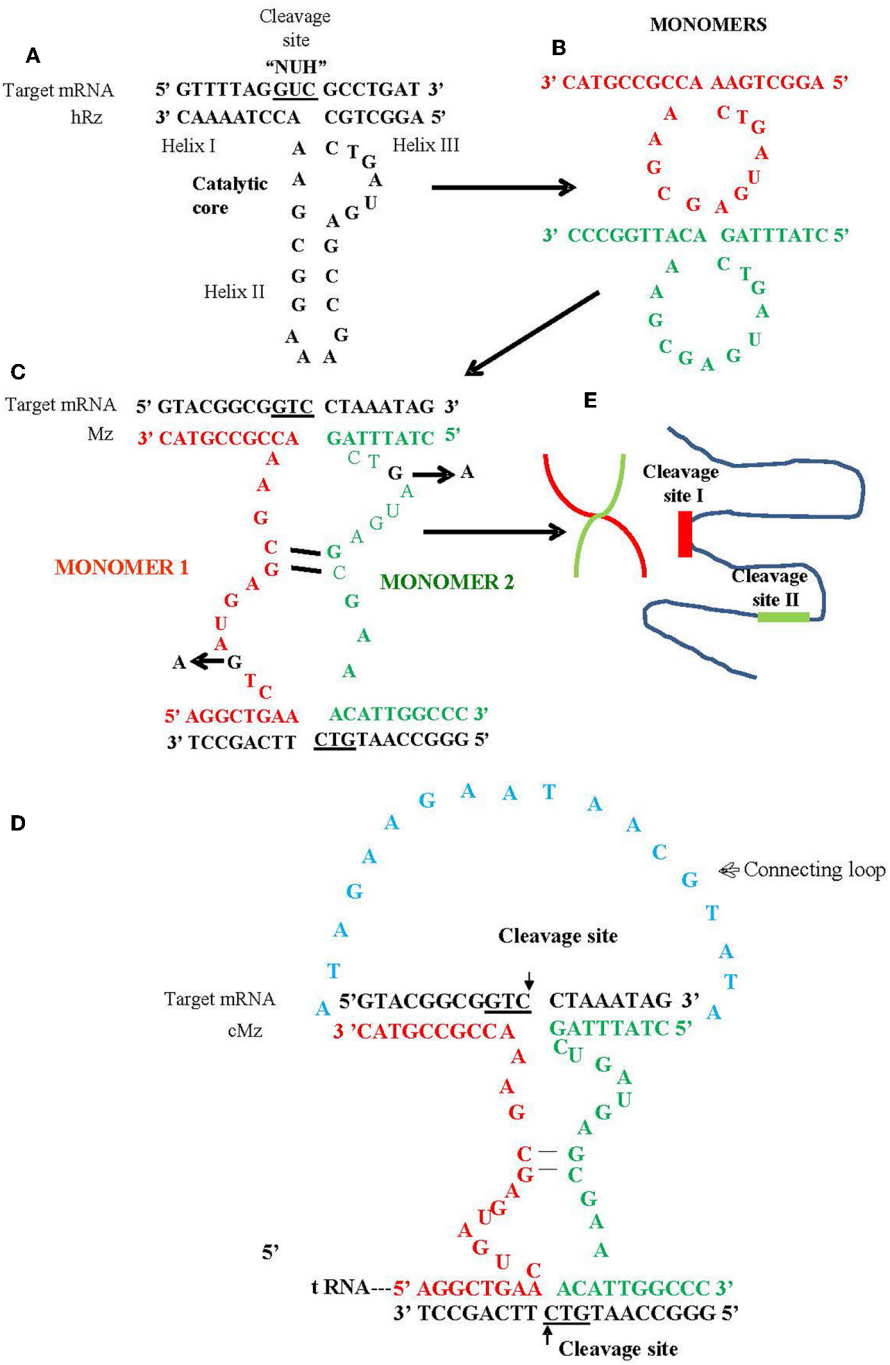
Schematic of hammerhead ribozyme (hRz) and maxizymes. **(A)** Structure of hRz. **(B)** Monomers derived from hRzs by deletion of helix II stem loop structure. **(C)** Monomers (Red or Green) undergoing bimolecular interaction leading to the formation of heterodimer/maxizyme, (guanine: G in Black) in the catalytic core for both monomers replaced by adenine (A) to generate inactive version of maxizyme **(D)** connected maxizyme with 13 nucleotide connecting chain (Blue) **(E)** Maxizyme binding to two different target sites on viral RNA.

In this study, we utilized a maxizyme approach to enhance the ribozyme activity and provide broad spectrum activity against escape variants (Haasnoot et al., 2007). Maxizymes (Mz) utilize minimized versions of two hRzs by combining them into a single catalytic unit (Kuwabara et al., 1998). A maxizyme consists of two minizymes (minimized hRz) that lack stem loop II of the hammerhead structure (Figure 1). While minizymes have lower catalytic activity compared to their parental hRzs, some have cleavage activity similar to or better than their parental hRzs when they are configured into a dimeric form such as maxizymes (Iyo et al., 2002, 2004; Kuwabara et al., 2002) (Figure 1).

Previous research has demonstrated that Mzs are catalytically more active compared to hRzs (Kuwabara et al., 1998, 2002; Hamada et al., 1999). The binding of one target site enhances unwinding of RNA secondary structures in the targeted RNA and serves as an alternative approach to the recruitment of RNA helicases, thereby cleaving less accessible sites in the target RNA molecule through binding two arms rather than one (Kuwabara et al., 2002).

Our maxizyme constructs demonstrated greater effectiveness against CHIKV infection than the hRzs they were derived from when expressed in both transformed cell cultures and transgenic mosquitoes. These results suggest that maxizyme can be an effective antiviral for arboviruses like CHIKV, DENV, and Zika as mosquito transgene effectors.

## Materials and methods

### Cells and viruses

African monkey kidney (Vero) cells (ATCC, USA) were maintained on Dulbecco's modified eagle medium (DMEM; Sigma Aldrich, USA) supplemented with 10% fetal bovine serum (FBS, Atlanta Biological, Flowery Branch, GA, USA) and non-essential amino acids [(1x), Gibco, USA)]. The CHIKV 181/25 strain is an attenuated vaccine strain (a gift from Dr. Scott Weaver, UTMB, Galveston) that was used for testing the effectiveness of our maxizymes *in vitro* and *in vivo*. We chose this strain for safety reasons since we do not have appropriate containment facilities for handling a virulent strain. Additionally, while the 181/25 strain is attenuated for human virulence, it does not exhibit significant reduction in mosquito infection.

### Design and cloning strategy for CHIKV maxizyme

We designed two versions of a CHIKV maxizyme, active Mz and inactive ΔMz, by combining the most effective anti-CHIKV hammerhead ribozymes (hRzs) #9 and #14 (Mishra et al., 2016). Each maxizyme version consists of two monomers, I and II, with the following components: tRNA^val^ promoter, partial target binding sites of hRz# 9 and #14, catalytic core (active: CTG, or inactive: CTA), and a termination signal (Figure 1 and Table 1).

**Table 1:** Sequence of maxizymes: each maxizyme sequence includes the Ae-tRNA ^val^ promoter (Black), monomer I (Red), monomer II (Green) and stop signal sequence (Purple).

**Ribozyme**	**Sequence**
**Maxizyme (Mz)**	ACCGTTGGTTTCCGTAGTGTAGTGGTTATCACGTCTGCTTCACACGCAGAAGGTCCCCGGTTCGAACCCGGGCACTACAAAAACCAACTTT AGGCTGAACTGATGACGCAAACCGCCGTACTTTTTTTACCGTTGGTTTCCGTAGTGTAGTGGTTATCACGTCTGCTTCACACGCAGAAGGTCCCCGGTTCGAACCCGGGCACTACAAAAACCAACTTT CTAATGACGCAAACATTGGCCCTTTTTTT
**Maxizyme (inactive) (ΔMz)**	ACCGTTGGTTTCCGTAGTGTAGTGGTTATCACGTCTGCTTCACACGCAGAAGGTCCCCGGTTCGAACCCGGGCACTACAAAAACCAACTTT AGGCTGAACTAATGACGCAAACCGCCGTACTTTTTTTACCGTTGGTTTCCGTAGTGTAGTGGTTATCACGTCTGCTTCACACGCAGAAGGTCCCCGGTTCGAACCCGGGCACTACAAAAACCAACTTT CTATTTAG CTAATGACGCAAACATTGGCCCTTTTTTT
**Maxizyme (cMz)**	ACCGTTGGTTTCCGTAGTGTAGTGGTTATCACGTCTGCTTCACACGCAGAAGGTCCCCGGTTCGAACCCGGGCACTACAAAAACCAACTTTAGGCTGAACTGATGACGCAAACCGCCGTACATAGAAGAATAACGTATACTATTTAGCTGATGACGCAAACATTGGCCCTTTTTTT

For the inactive maxizyme, the guanine in the catalytic core was replaced by adenine (A) nucleotide underlined. For the connected maxizyme both Monomer I and Monomer II are connected via a connecting loop (Blue) expressed under a single Ae-tRNA ^val^ promoter.

The active Mz was cloned into an Aedes expression lentivirus plasmid, pLAeARH (Nawtaisong et al., 2009), in two steps. The first step amplified Mz monomer I from the pLAeRz#9ARH vector along with the promoter and termination signals using PCR primers Mz-I F and Mz-I R (Table 1) and cloned the amplified sequence into *Bam HI* and *Not* I sites of pLAeARH. In the second step, the Mz-II was amplified from the Mz-II template using the primers Mz-II F and Mz-II R (Table 2), and cloned into *Pme* I and *Not* I sites of the lentivirus plasmid pLAeMzIARH downstream of Mz-I to construct pLAeMzIAeMzIIARH.

**Table 2:** Primers/oligos used for the construction of anti-CHIKV maxizyme and connected maxizyme, and for confirmation of maxizyme expression.

**Name**	**Sequence (5'-3')**
Mz-I F	TTTTTTTTTGGATCCACGGATCCTCTAGACCGTTGGA
Mz-I R	AATGCATGAGCGGCCGCGTTTAAACAAAAAAAGTACGGCGGTTTCGCTCATCAGTTCAGCCTTTGTTGGTTTTTGTAGTGCCCG
Mz-II	CGCTCATCAGCTAAATAGTTGTTGGTTTTTGTAGTGCCCGGGTTCGAACCGGGGACCTTCTGCGTGTGAAGCAGACGTG
Mz-II F	ATATACGTGTTTAAACACCGTTGGTTTCCGTAGTGTAGTGGTTATCACGTCTGCTTCACACGC
Mz-II R	TGATGCTGAGCGGCCGCAAAAAAAGGGGCCAATGTTTCGCTCATCAGCTAAATAGTTG
ΔMz-I R	AATGCATGAGCGGCCGCGTTTAAACAAAAAAAGTACGGCGGTTTCGCTCATTAGTTCAGCCTTTGTTGGTTTTTGTAGTGCCCG
ΔMz-II R	TGATGCTGAGCGGCCGCAAAAAAAGGGGCCAATGTTTCGCTCATAAGCTAAATAGTTG.
ΔMz-II	CGCTCATAAGCTAAATAGTTGTTGGTTTTTGTAGTGCCCGGGTTCGAACCGGGGACCTTCTGCGTGTGAAGCAGACGTG
cMz-sense	AAACAGGCTGAACTGATGACGCAAACCGCCGTAC ATAGAAGAATAACGTATACTATTTAGCTGATGACGCAAACATTGGCCCTTTTTTTGC
cMz-antisense	GGCCGCAAAAAAAGGGCCAATGTTTGCGTCATCAGCTAAATAGTATACGTTATTCTTCTATGTACGGCGGTTTGCGTCATCAGTTCAGCCTGTTT
Common tRNA F	ACCGTTGGTTTCCGTAGTGTAGTG
hRz#9 R	ATAAGAATGCGGCCGCGTTTAACGTACGGCGGTTTCGGCCTTTCG
hRz#14 R	ATAAGAATGCGGCCGCGTTTAACGGGCCAATGTTTCGGCCTTTC
Mz-II R	GCCAATGTTTGCGTCATCAGC
ΔMz-MII (inactive)	TGATGCTGAGCGGCCGCAAAAAAAGGGGCCAATGTTTCGCTCATAAGCTAAATAGTTG
Ae-tRNA ^val^ F	TTTTTTTTTTGTCGACACCGTTGGTTTCCGTAGTGTAG
Ae-tRNA ^val^ R	TTTTTTTTTGCGGCCGCGTTTAAACTCTAGAAAAGTTGGTTTTTGTAGTGCCC
Transgene F	TTTAAATTTCCGCGGACCGTTGGTTTCCGTAGTGTAGTGG
Transgene R	TTTAAATTTAGATCTTGAGGGGATCTGCGGCCG
Poly A R	TATATCCTGAGGGGATCTGCG

The inactive ΔMz was made by introduction of a point mutation (CTG-CTA) to disrupt the catalytic activity of the maxizyme. Cloning of this inactive ΔMz involved generation of the ΔMz monomer I from the pLAeMzIARH vector with active monomer I through PCR amplification using Mz-I F and ΔMz R primers (Table 2). The amplified sequence was inserted at the restriction sites *Bam HI* and *Not* I pLAeARH, as described above. The remaining portion of inactive ΔMz was created by amplification from the template ΔMz monomer II using primers Mz-II F and ΔMz II R MI (Table 2) and cloned into *Pme* I and *Not* I sites of the lentivirus plasmid pLAeΔMzIARH. The final vector was pLAeΔMzIAeΔMzIIARH. Finally, a CMV-ds RED fluorescent marker was cloned into both the maxizyme plasmids, as previously described (Mishra et al., 2016).

### Generation of clonal cell populations

Vero cells were seeded into 6 well plates and 24 hours (hrs) later the ribozyme expression plasmids were transfected using lipofectamine LTX and plus reagent (Invitrogen, USA), following the manufacturer's instructions. Forty-eight hrs later, the transfected cells were selected using 200 μg/ml of hygromycin B (Invitrogen, USA) and maintained for two passages before sorting into 96 well plates. Cell sorting and screening was performed as previously described (Mishra et al., 2016).

### RT-PCR based detection of ribozyme expression

Total cellular RNA was TRIzol-extracted following the manufacturer's protocol (Invitrogen, USA). The concentration of extracted RNA was determined spectrophotometrically using a NanoDrop ND-1000 UV-Vis Spectrophotometer. A total of 5μg of RNA was treated with Turbo DNase I (Ambion, USA) and directly used for reverse transcriptase (RT) positive and negative reactions using the Superscript III one step RT-PCR kit (Invitrogen, USA). For the RT negative reaction, Taq DNA polymerase (Invitrogen) was used. A common Ae-tRNA ^val^ forward primer was used along with ribozyme-specific reverse primers (Table 2). The RT-PCR products were resolved on 2.0% agarose gels (Ethidium bromide concentration 10 mg/ml) at 105 V for 1 h. Similarly, RT-PCR was performed using twenty mosquitoes per reaction to check for the expression of maxizyme in transgenic mosquitoes.

For mosquito analysis, we collected mosquitoes and organized them into groups of 20. We manually homogenized these groups in 500 μL of Trizol (Invitrogen, USA) followed by centrifugation at 12, 000 g for 10 min at 4°C. After centrifugation, we processed the resulting supernatant for RNA extraction, following the manufacturer's instructions (Invitrogen, USA).

### CHIKV infection of vero cells

Wild-type Vero cells and selected clonal Vero cells expressing effector molecules specific to CHIKV were plated at a density of 1 × 10^5^ cells per well. After overnight incubation at 37 °C, the cells were washed once with serum-free DMEM and were challenged with CHIKV 181/25 at an MOI of 0.05 or 0.5 for 2 h. The infected cells were fed with fresh DMEM supplemented with 10% FBS. Two days post infection (dpi) supernatants were collected for TCID_50_, RT-qPCR, and caspase 3 assays.

### TCID_50_-IFA analysis

CHIKV cell supernatants were collected at 2 dpi for assay. Briefly, 10-fold serial dilutions of virus supernatant were prepared in DMEM plus 10% FBS, and 100 μl of each dilution was aliquoted into 10 wells of a 96 well plate pre-seeded with 1 × 10^5^ cells per well. After 3 dpi, the plates were fixed and stained with a primary antibody (1:100) specific to CHIKV capsid protein (Virostat, USA). Infected positive cells were recognized using a biotinylated secondary antibody (GE healthcare) and streptavidin detection system (Invitrogen). An inverted fluorescent microscope (Nikon, Japan) was used for observation of cytoplasmic fluorescence. Wells scored positive for the presence of green cytoplasmic fluorescence. The numbers of positive wells were counted and the virus titers calculated according to Karber's method (Kärber, 1931). The titer was expressed as log_10_TCID_50_/ml.

### Caspase 3 assay

The caspase assay was performed using the Caspase-glo 3/7 kit (Promega, USA) according to the manufacturer protocol. Vero cells were plated in 96 well plates 24 h before infection. The cells were then infected with the clones exhibiting complete suppression at an MOI of 0.05. At 2dpi, the cells were incubated with Caspase-glo reagent for 1 h in the dark at room temperature. The caspase activity was measured by detecting the luminescence using LMAX-2 luminometer (Molecular Devices).

### Quantitative real time PCR

Viral RNA was isolated from 2 dpi supernatants collected from clones exhibiting complete suppression at an MOI of 0.05 for CHIKV, using the viral RNA mini kit (Qiagen, Germany). All isolated nucleic acids were quantitated using a Nanodrop ND-1000 spectrophotometer (Thermofisher). Stock virus with known titer was used as a control to generate the standard curve. Complementary DNA synthesis was carried out using the Gene Amp RNA PCR MULV reverse transcriptase kit (Applied Biosystem) both for samples and standards. For CHIKV, the primer targeting the nsP2 region of the virus, nsP2 reverse: aaattcggcctgaaccttct, was utilized (Ho et al., 2010). One cycle of 30 minutes at 42°C and 5 min at 99°C was performed (Mishra et al., 2016). The absolute quantification was performed on the 7500 fast real-time PCR system (Applied Biosystem) using Power sybr green master mix (Applied Biosystem) at a particular thermocyclic condition of one cycle for 2 min at 50°C, one cycle for 10 min at 95°C, 40 cycles for 15 s at 95°C, and 1 min at 60°C. For quantification of CHIKV, the above mentioned primer nsP2 reverse along with nsP2 forward: ttctgggggtcagagaaaga was used (Ho et al., 2010). Beta-actin was used as an internal control for all RT-qPCR assays. The slope of the standard curve was −2.8 and the R^2^ value was 0.97. The absolute quantification of viral RNA copies/ml in the samples was performed by comparing them to the corresponding standards with known viral titer.

### Construction of connected maxizyme-expressing transgenes in the *piggyBac* vector and mosquito injections

For the generation of transgenic mosquitoes expressing maxizymes, we adopted the connected maxizyme approach. In this approach, both monomers of the CHIKV-Mz were connected using a 13-nucleotide long linker sequence (atagaagaataacgtata) and expressed using a single Ae-tRNA^val^ promoter (Figure 1). This was done to increase the efficiency of formation of a bi-molecular heterodimeric maxizyme structure (Hamada et al., 1999; Kuwabara et al., 2002) and reduce the chance of inactive homodimer formation. The cloning of the transgene into the *piggyBac* vector, pXL-BacII-3xP3-ECFP, involved PCR amplification of the Ae-tRNA ^val^ pol III promoter from the pLAeARz#9RH vector using the Ae-tRNA ^val^ F and R primers (Table 2) and inserting it into the pLAeARH plasmid *Sal* I and *Not* I sites. The complementary oligonucleotides of connected maxizyme (cMz-sense and cMz-antisense, Table 2) were annealed together and cloned into the *Bam* HI and *Not* I sites downstream of the promoter in pLAeARH, resulting in the plasmid pLAeAcMzRH. The connected Mz and expression units were PCR amplified from the pLAeAcMzRH vector using transgene F and R (Table 2) primers and subcloned into the *Sac* II and *Bgl* II sites of the *piggyBac* vector.

### Generation of transgenic mosquitoes and identification of transgene integration sites using splinkertte PCR

Transgenic mosquitoes were generated by embryo injection of *piggBac* cMz expression vectors into mosquito embryos, essentially as previously described (Mishra et al., 2016). Higgs White Eye (HWE) mosquitoes were used in this study, facilitating detection of the 3 × P3-ECFP eye-specific transgene marker gene and allowing transgenic mosquitoes to be detected from UV fluorescence in the eyes. Mosquitoes were reared in an Arthropod containment level-2 (ACL-2) facility at 28^o^C with 60–80% relative humidity. They were maintained on 10% sucrose solution and water and artificially fed with citrated sheep's blood (Colorado Serum Company, Denver, CO, USA) and 1 mM/10 ml of phagostimulant ATP. During infections, virus-infected cell culture medium was mixed with an equal volume of feeding solution.

A total of five independent transgenic lines were established and a splinkerette protocol was adopted from Potter and Luo (2010) to assess the genomic location of each *piggyBac*-integrated transgene as previously described (Mishra et al., 2016). All five transgenic mosquito lines exhibited unique integration sites in *Ae. aegypti* genome (Table 3).

**Table 3:** Location of transgene in the mosquito genome.

**Transgenic line**	**Chromosome number**	**Supercontig number**
CMCM1	3q	1.13
CMCM46	No	1.648
CMCM75	No	1.891
CMCF4	No	1.187
CMCM80	No	1.1431

The transgene integration sites in chromosomes and supercontigs of Aedes mosquitoes. 3q-chromosome number 3; no-chromosome number not known.

### Analysis of CHIKV infection in cMz transgenic mosquitoes

Control and transgenic lines were fed infectious blood meals with a viral titer of 3 × 10^9^ TCID_50_/mL. Both the infected controls and cMz transgenics were maintained for seven days on 10% sucrose solution prior to feeding infected bloodmeals. For each transgenic line, a total of 15 mosquitoes in small containers were allowed to feed for 2 h on 700 μL probing solution (50% FBS (164 mM) + NaCl (100 mM) + NaHCO_3_ (0.2 mM) + ATP (50 μg) + sucrose, pH 7.0) contained between two parafilm membranes, as described by Franz et al. (2006). Successful feeding was confirmed by observation of fully engorged mosquitoes in all groups. Mosquito homogenates were processed as previously described (Mishra et al., 2016).

### Statistical analysis

The statistical analysis performed in this study is described in figure legends. All the statistical tests were carried out using GraphPad Prism version 9.3.0.3.

## Results

### Effect of maxizyme on CHIKV replication *in vitro*

To compare the effect of our maxizyme on CHIKV replication, we successfully constructed two lentivirus expression constructs with maxizyme: Mz, and ΔMz. Then, we transformed these maxizyme expression plasmids along with plasmids expressing the parental hRz #9 and # 14 and isolated several clonal cell populations for each. All these clonal populations were challenged with CHIKV at two different MOIs, and the effect was evaluated by the presence or absence of CPE. Our screening results revealed clones #2 and #15 of Mz, clones #5 and #27 of hRz#9, and clone #37 of hRz#14 were resistant to CHIKV CPE. However, all the clones from the inactive ΔMz had marked CPE (data not shown).

**Figure 2:**
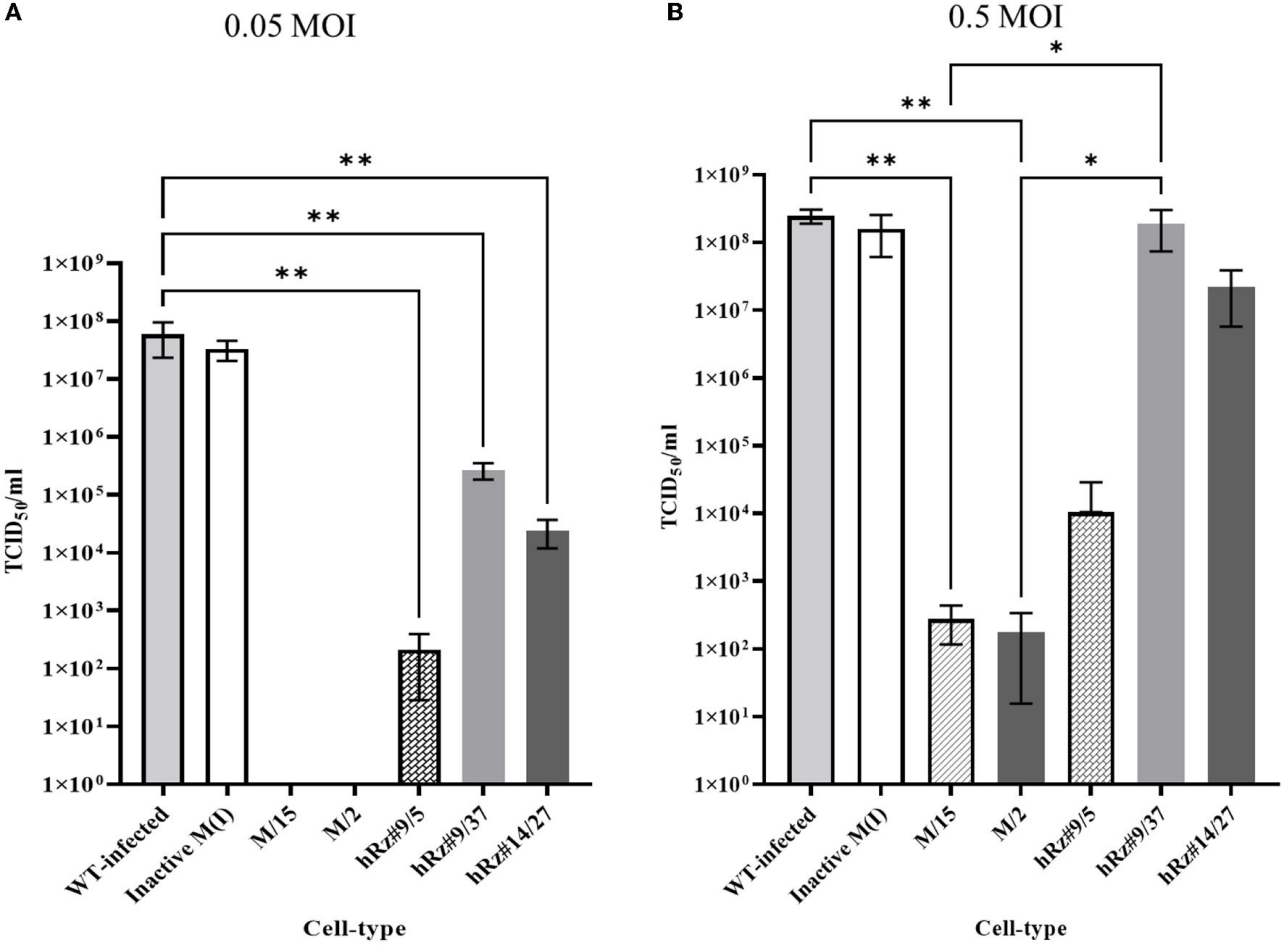
TCID_50_-IFA analysis of clonal populations expressing CHIKV specific maxizymes and hammerhead ribozymes with two different MOI. **(A)** Challenge MOI of 0.05 and **(B)** Challenge MOI of 0.5, cells were fixed and stained with anti-CHIKV capsid specific antibody 3 dpi. WT: Untransformed wild type Vero cells and M (I): inactive control for maxizyme. Each clone is represented as maxizyme or hRz number / clone number. Each bar represents an average CHIKV titer from three independent experiments. Error bars represent the standard deviation among the three independent replicates for each clone. Statistical analysis was performed using the Two-way ANOVA test and Tukey's multiple comparisons test (* *p* < 0.05,** *p* < 0.005).

**Figure 3:**
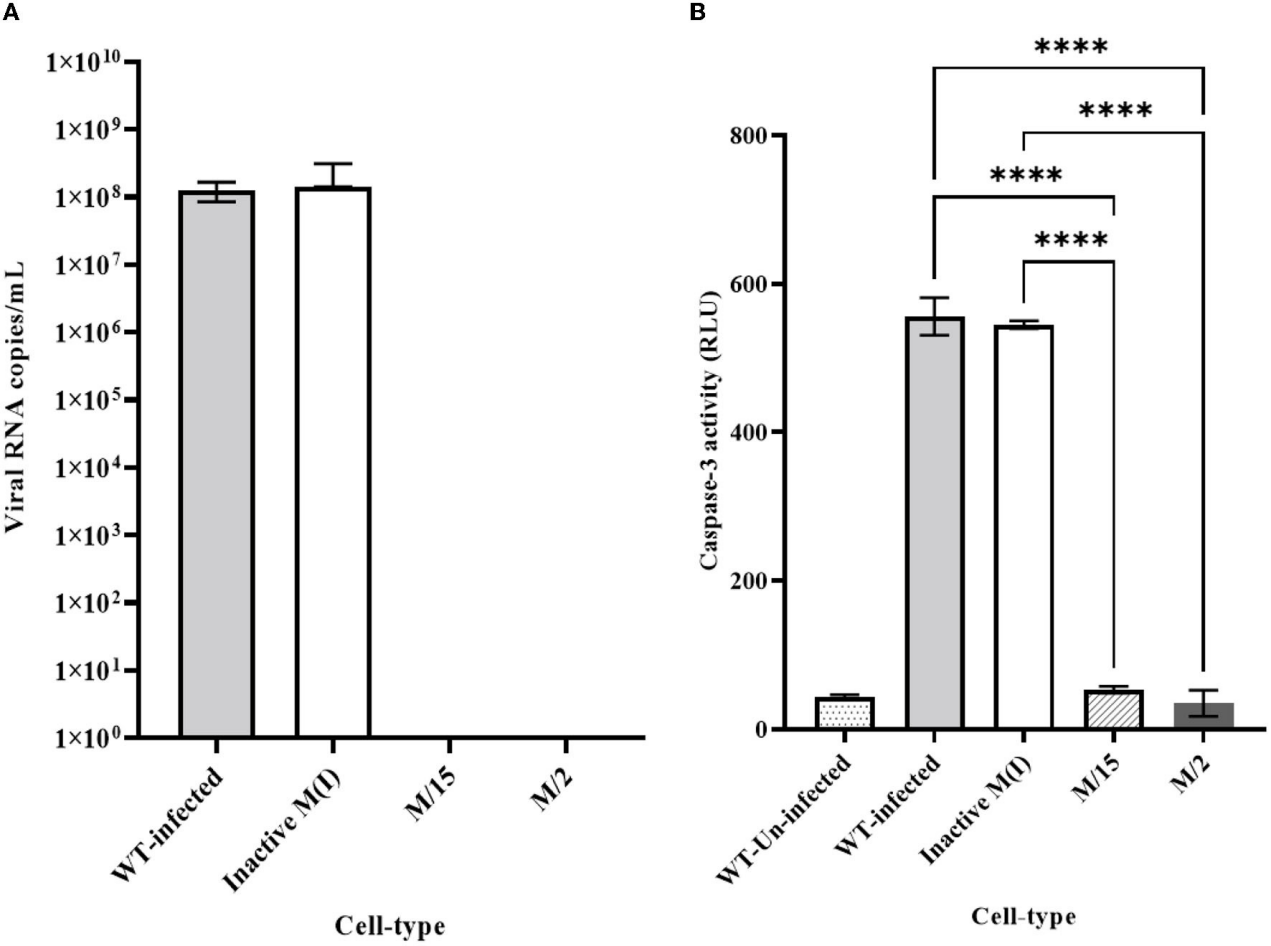
Quantitative RT-qPCR and caspase 3 analysis for clonal expressing antiviral ribozymes specific to CHIKV. **(A)** RT-qPCR performed on viral RNA isolated from the infected cell culture supernatants collected 2 dpi challenged at an MOI of 0.05. nsP2 specific primer were employed for quantification. **(B)** Caspase 3 analysis performed by infecting healthy control and transformed clonal cells using supernatant collected from 2dpi challenge experiment at an MOI of 0.05. WT designates untransformed Vero cells, either infected with virus as the infected control, or incubated with media as the Unifected control. M-maxizyme, M(I)- maxizyme Inactive, each clone is represented as maxizyme/clone number. Each bar is an average of three independent infection experiments. Bars denote the standard deviation among the three independent replicates done for each clone. Statistical analysis was performed using the Two-way ANOVA test and Tukey's multiple comparisons test (**** *p* < 0.0001).

We employed two different MOIs in our study to more thoroughly assess the effectiveness of maxizyme against CHIKV replication. At a lower MOI (0.05), the Mz clones #M2 and #M15 showed eight logs of CHIKV suppression as compared to the negative controls: i.e., untransformed wild-type and ΔMz transformed Vero cells (Figure 2A). However, the hRzs #9/5, #9/37, and #14/27clones showed six and three logs of suppression relative to the negative controls (Figure 2A). We also determined the CHIKV viral RNA copies by RT-qPCR assay in the supernatant collected of the infected maxizyme clones. The results revealed that Mz clones M/2 and M/15 completely suppressed viral RNA production, unlike the negative controls (Figure 3A). Additionally, we performed a caspase-3 assay to measure the virus-induced apoptosis. The infected virus supernatant collected from the Mz clones was tested for caspase-3 activity and the supernatants of both Mz clones exhibited caspase-3 activity similar to the levels of uninfected Vero cells (Figure 3B). However, the supernatant of the negative control, ΔMz, showed 500 times more activity than uninfected controls and Mz clones (Figure 3B), reflecting no effect on CHIKV replication.

**Figure 4:**
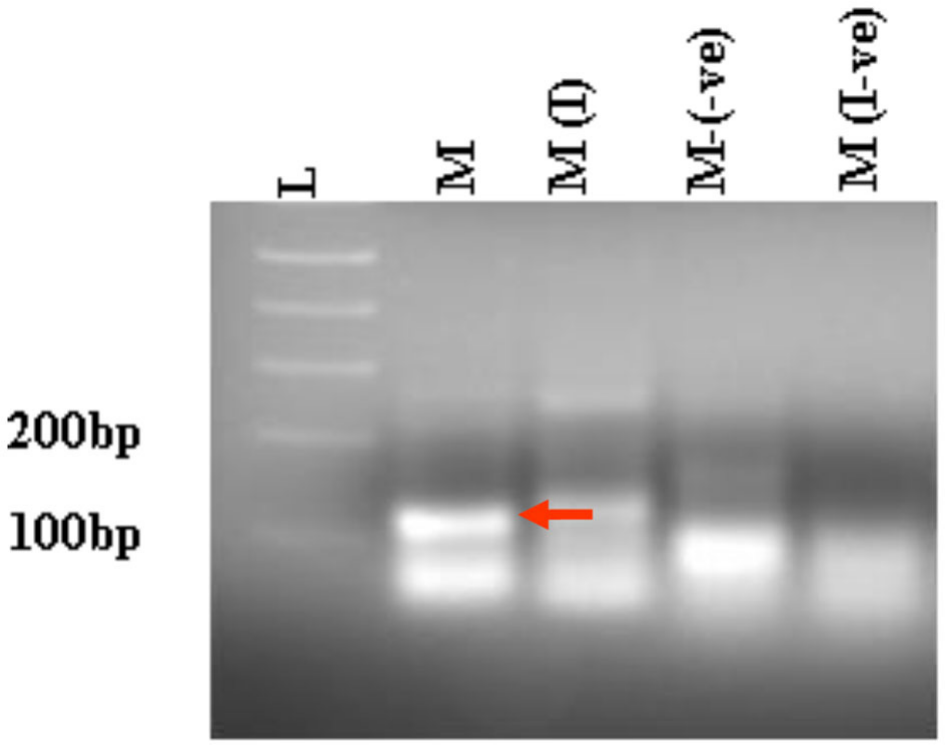
RT-PCR analysis of selected clonal cell populations expressing antiviral ribozyme constructs specific to CHIKV. The presence of the desired band specific to each CHIKV maxizyme is evident, where M: active maxizyme, M (I): inactive maxizymes, M(-ve) and M(I-ve) is RT-negative reaction. The presence of the correct band (110 bp size) is indicated by the red arrow. L- 1kb plus DNA ladder.

Next, we compared the level of CHIKV suppression for the hRz and Mz clones at a higher MOI of 0.5. Our initial tests at a lower MOI of 0.05 demonstrated that the maxizyme was highly effective in suppressing CHIKV replication. However, it was important for us to understand how this maxizyme performed across a range of virus concentrations, as this reflects the diverse conditions found in the field. The results revealed the Mz clones # M5 and # M2 suppressed CHIKV replication by six logs as compared to the negative controls. However, the suppression level of hRz clones #9/5, #9/37, and #14/27 clones dropped to four, zero and half a log, respectively. The maxizyme expression in the clones was confirmed by RT-PCR (Figure 4). Overall, under *in vitro* conditions, the maxizyme was more effective in suppressing CHIKV replication than the parental hammerhead ribozymes.

### Effect of maxizyme on CHIKV replication in transgenic mosquitoes

**Figure 5:**
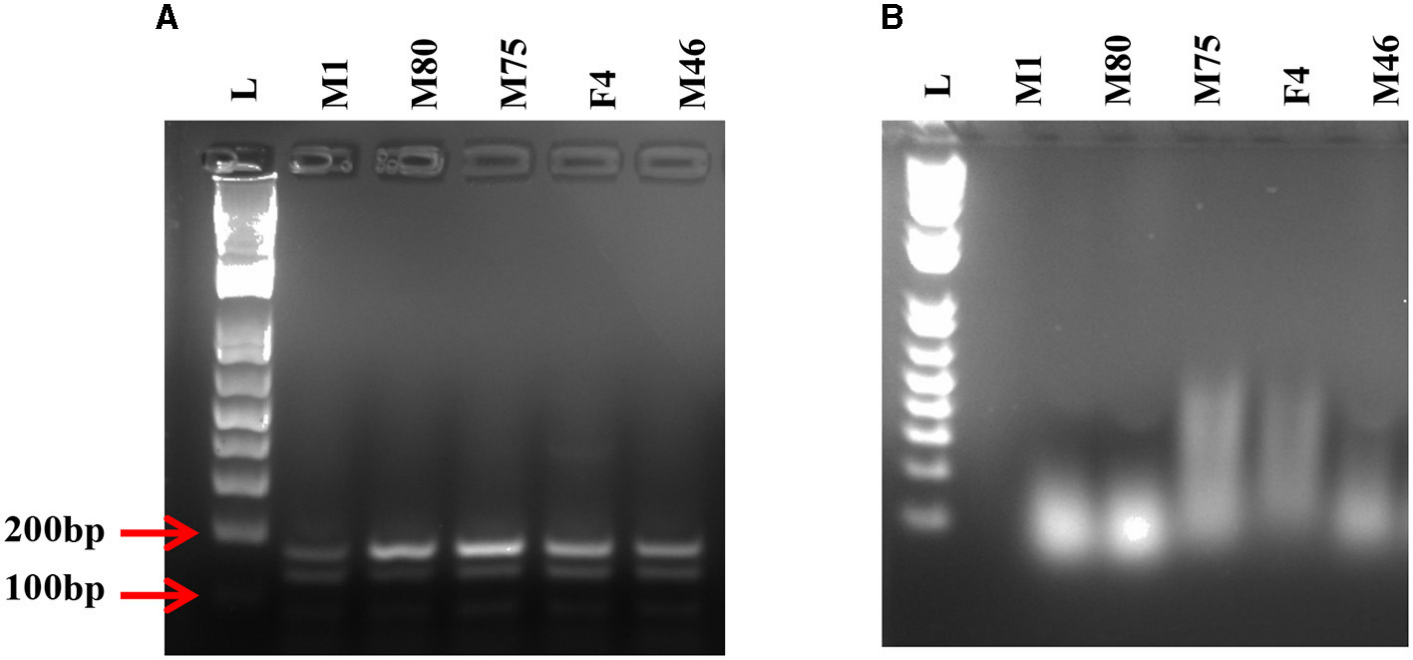
RT-PCR on transgenic mosquitoes. **(A)** Agarose gel displays the presence of desired 170 bp band confirming the expression of the connected maxizyme in each transgenic mosquito line. **(B)** RT negative reaction run using Taq polymerase shows the absence of the desired band. L- 1kb plus DNA ladder and M1, M80, M75, F4 and M46 are transgenic lines.

To test the effectiveness of Mz in controlling CHIKV transmission, we generated five transgenic lines of *Ae. aegypti* mosquitoes. For transgenesis, we used a connected maxizyme (cMz) approach for ease of integration into *piggyBac* vector (pXL-Bac-II-ECFP) and to increase the efficiency of formation of active heterodimeric structures. The pXL-Bac-II-ECFP-cMz expression and transposase helper plasmids were co-injected into *Ae. aegypti* embryos as previously described (Mishra et al., 2016), and the transformation efficiency ranged from 0.5 to 20% (Table 4). A percentage fluorescence of 57–96% was obtained from all the transgenic lines at G5 (data not shown). Generation 5 positive mosquitoes were then backcrossed to wild-type HWE mosquitoes as previously described (Mishra et al., 2016) to generate a heterozygous G6 transgenic mosquito population. cMz expression in these transgenic lines was confirmed by RT-PCR (Figure 5). Each of the integration sites of these five lines were identified in the mosquito genome at different super contigs (Table 3).

**Table 4:** Percentage transformation frequency for cMz containing transgenic lines.

**Transgenic lines**	**Total screened**	**Positives**	**% transformation**
CMCM1	340	11	3.2
CMCF4	299	4	1.3
CMCM80	252	7	2.7
CMCM46	108	21	20
CMCM75	612	3	0.5

Screening for transgenic mosquitoes was performed based upon eye specific cyan fluorescent protein.

### Challenge of transgenic mosquitoes with CHIKV

**Figure 6:**
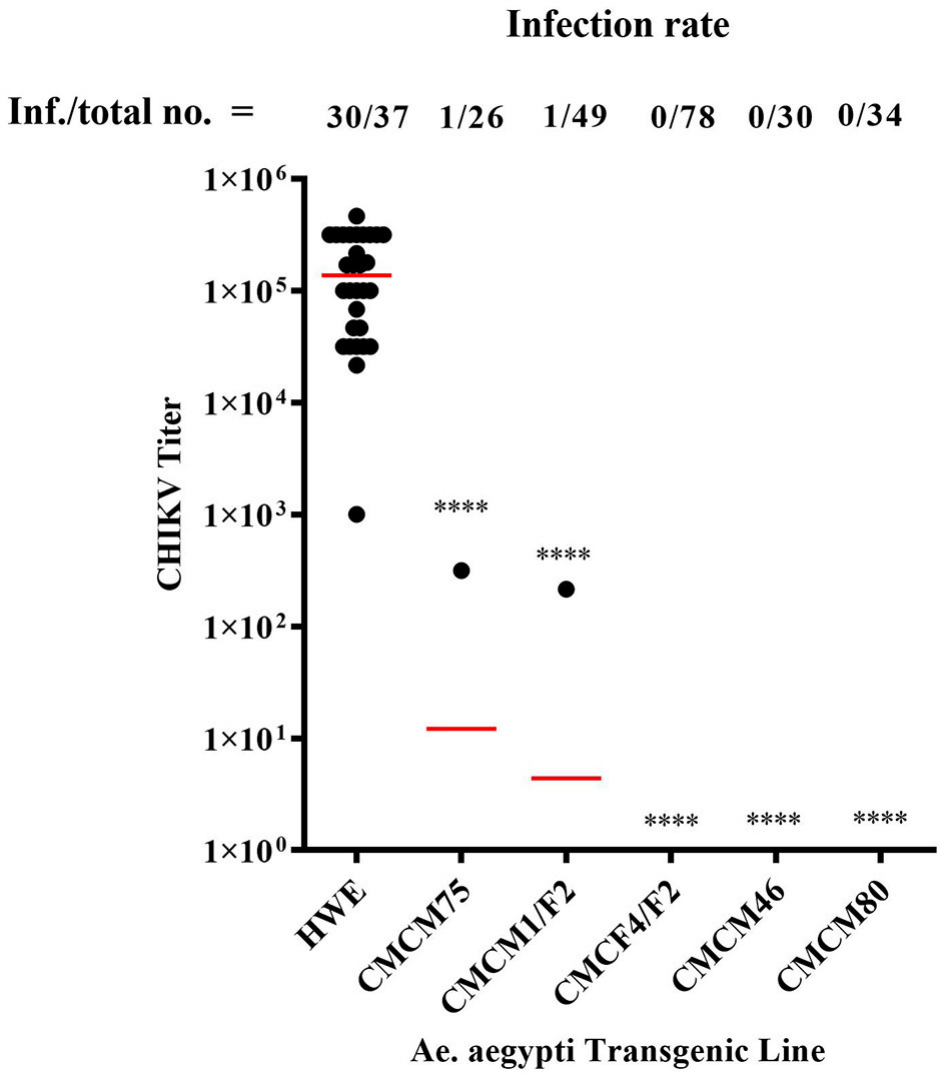
TCID_50_- IFA performed on CHIKV challenged transgenic G6 heterozygous mosquito population. TCID_50_-IFA was performed on homogenates of single transgenic mosquito fed an infectious blood meal compared to control HWE mosquitoes. Analysis was performed seven days post infectious blood meal. “Inf.” indicates the number of infected mosquitoes analyzed and “total no.” indicates the total number of mosquitoes analyzed. HWE-Higgs White Eye mosquitoes, CMCM75, CMCM1/F2, CMCF4/F2, CMCM46 and CMCM80 are connected maxizyme expressing transgenic mosquitoes. Statistical analysis was performed using the Two-way ANOVA test and Tukey's multiple comparisons test. All the transgenic lines had a statistical significance of ****p < 0.0001 as compared to the HWE line.

Heterozygous G6 transgenic lines were challenged with CHIKV by oral exposure to infectious CHIKV blood meal, as previously described (Mishra et al., 2016). Mosquitoes were collected at 7 days post-blood meal (7 dpbm) and individual homogenates were made of each mosquito in 200 μl of DMEM. Homogenates were filtered through a 0.2 μM membrane filter for further analysis. Mosquitoes from three of the lines, CMCM80, CMCM46, and CMCF4/F2, exhibited complete suppression and had a 0% infection rate. However, non-transgenic (wild-type) HWE mosquitoes had an infection rate of 81 % with an average infectious virus titre of 2 × 10^5^ TCID_50_/ml (Figure 6). In contrast, the transgenic mosquito lines CMCM75 and CMCM1/F2 had an infection rate of 3.8 and 2 %, and infectious virus titer of 3.2 × 10^2^ and 2.2 × 10^2^ TCID_50_/ml, respectively (Figure 6).

**Figure 7:**
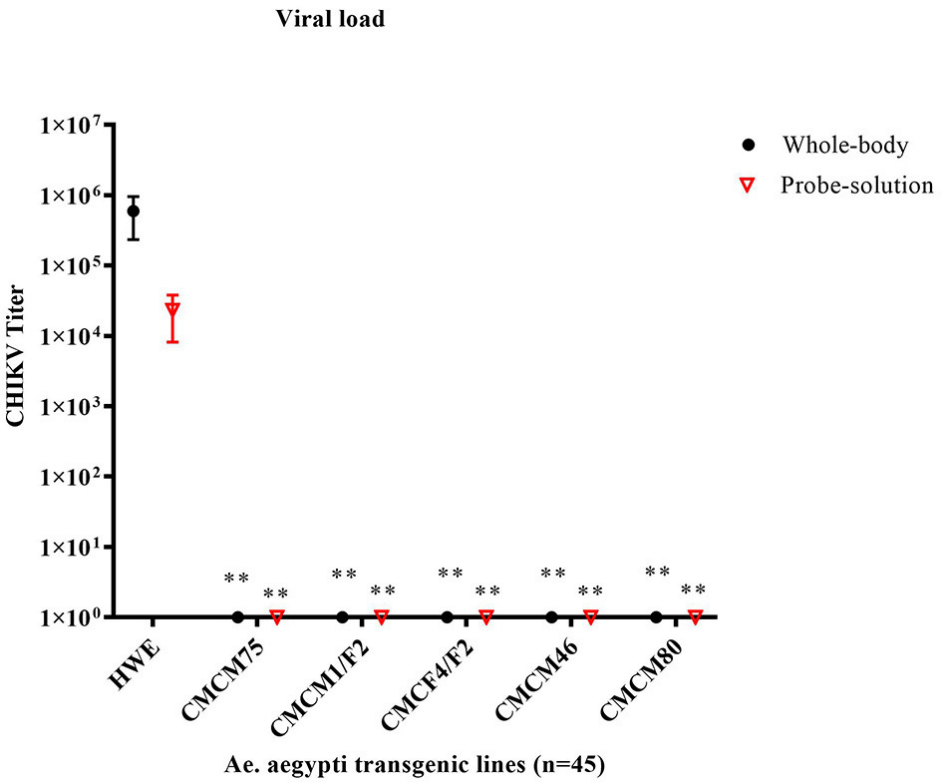
TCID_50_- IFA performed on the probe solution (saliva) and whole-body of CHIKV challenged transgenic G6 heterozygous mosquito population. Briefly, pooled probe solutions and whole-body homogenates were collected from the infected transgenic and infected wild-type mosquitoes at 7dpbm. The virus titer of these samples was determined by TCID_50_-IFA. Statistical analysis was performed using the Two-way ANOVA test and Tukey's multiple comparisons test. All the transgenic lines (whole body and probe solution) had a statistical significance of **p < 0.005 as compared to the HWE line.

Next, we tested the transmission potential of these transgenic mosquitoes. As expected, the wild-type HWE mosquitoes effectively transmitted CHIKV to probe solution with an average titer of 2.3 × 10^4^ TCID_50_/ml, along with an average whole-body titer of 5.9 × 10^5^ TCID_50_/ml (Figure 7). However, no infectious virus was detected from either the probe solution or the whole-body homogenates of the five transgenic mosquito lines (Figure 7). Overall, these results indicate that the transgenic mosquito line expressing cMz completely inhibited CHIKV infection and transmission and did so from a heterozygous genetic background.

## Discussion

Anti-pathogen effectors have been investigated for several arboviruses such as CHIKV (Mishra et al., 2016), DENV-2 (Franz et al., 2006), DENV3 and CHIKV (Yen et al., 2018), and Zika (Buchman et al., 2019). The presumptive outcome of such investigations is the development of transgenic mosquitoes that could eventually be used for replacement of naturally arboviruses competent mosquitoes (Marshall et al., 2019). Thus, far, researchers have come close to releasing some of these transgenic approaches, but the caveat remaining is the difficulty of introgression into wild-type mosquitoes due to possible position effects on efficient expression, the presence of multiple strains of virus circulating in the target areas, and the size of the transgene. *Wolbachia* infection of mosquitoes has been successful at controlling arboviruses in various countries, however the stability and the effect of such use is not yet clear (Yen and Failloux, 2020). To overcome these limitations, we have developed a strategy of using maxizymes, which has the potential advantages of small size transgene, higher potency, effectiveness against escape variants, and possible adaptability to target multiple arboviruses simultaneously.

We first demonstrated the effectiveness of our maxizyme in inhibiting CHIKV replication by measuring virus production, viral RNA, and virus-induced apoptosis in Vero cells. To mimic the variability of virus doses in nature we tested the effectiveness of Mz under lower (0.05 MOI) and higher doses (0.5 MOI). The Mz was effective at inhibiting CHIKV replication at both doses of virus as compared to the parental hRzs. The observation two 2 logs of virus production at higher MOIs in Mz clones could be due to higher doses of the virus skewing the target-enzyme ratio, or it could be due to weaker expression of both the monomers (Figure 2). Nevertheless, the CHIKV suppression was more significant than the controls and parental hRzs (Mishra et al., 2016). Overall, Mz showed a higher potency and effectiveness in controlling CHIKV replication as compared to the parental counterparts *in vitro*.

We utilized a connected maxizyme in the transgenic mosquitoes owing to the advantages discussed in the results section. All five transgenic lines were highly refractory to CHIKV infection and prevented virus transmission, and this was true in a heterozygous state. This latter observation is important because if we envision release of these transgenes in a population control strategy, heterozygotes will predominate in the first and subsequent generations. Additionally, this approach is not susceptible to gene position effects, as demonstrated by cMz expression and CHIKV inhibition irrespective of the integration loci (Table 4).

Although these results are promising, our study has the following limitations. First, we have not tested the effectiveness of Mz against a virulent strain of CHIKV due to the unavailability of BSL-3 facility. While the 181/25 CHIKV strain is attenuated for human infection, and lacks the A226V mutation attributed to recent epidemic outbreaks of CHIKV, the maxizyme targets we chose are present in both the attenuated and virulent strains. Additionally, based upon our results in this and prior studies, infection of cell cultures and mosquitoes is not appreciably affected by the dual mutations in the E2 protein responsible for the attenuation in humans (Gorchakov et al., 2012). Second, the stability of the transgene and its effectiveness over generations in our transgenic mosquitoes was not addressed. This will take additional time and analyses, and we will pursue this in our future studies.

These results confirm that maxizymes can be potent inhibitors of CHIKV replication in mammalian cells or transmission in transgenic mosquitoes. Additionally, we could use this approach to design Mz against multiple arboviruses and possibly develop a universal transgenic mosquito resistant to several arboviruses (Carter et al., 2015).

## Data availability statement

The original contributions presented in the study are included in the article/supplementary material, further inquiries can be directed to the corresponding author.

## Ethics statement

Ethical approval was not required for the studies on animals in accordance with the local legislation and institutional requirements because only commercially available established cell lines were used.

## Author contributions

PM: Conceptualization, Investigations, Validation, Resources, Data curation, Formal analysis, Methodology, Writing—original draft, Writing—review & editing. VB: Resources, Writing—original draft, Writing—review & editing. MF: Conceptualization, Funding acquisition, Resources, Supervision, Writing—review & editing.
